# Color Stability of Different Aesthetic Arch Wires in Beverages Consumed During the COVID-19 Era: An In Vitro Study

**DOI:** 10.7759/cureus.50542

**Published:** 2023-12-14

**Authors:** Sharvari S Khedkar, Usha Shenoy, Ananya Hazare, Himija Karia, Pritam Khorgade, Nivedita Nandeshwar, Sangeeta Bhattacharya

**Affiliations:** 1 Department of Orthodontics, VSPM (Vidya Shikshan Prasarak Mandals) Dental College and Research Centre, Nagpur, IND

**Keywords:** covid-19 pandemic, dental esthetics, orthodontic treatment dental esthetics, beverages, color stability, orthodontic arch wire

## Abstract

Introduction

One of the prime reasons for patients seeking orthodontic treatment is improvement in their aesthetics or appearance. With a greater number of adult patients now opting for orthodontic treatment, the demand for aesthetic orthodontic materials has increased. With the background of the recent coronavirus disease 2019 (COVID-19) pandemic and the popular role of strongly pigmented beverages that play an immunity-boosting role, studies exploring the effect of such beverages on orthodontic appliances may improve the decision-making process of selecting such aesthetic appliances.

Materials and methods

Four brands of wires and six beverages were included in this study. The wires were Teflon-, epoxy-, or ceramic-coated. Convenience sampling was done, and five samples of each brand were prepared to be tested in each solution. Samples were tested under a spectrophotometer after immersing them in various solutions for two, four, and eight weeks. A comparison of aesthetic degradation due to color changes amongst four brands of archwires was done by applying the one-way analysis of variance (ANOVA) test. P values were calculated for all samples to determine whether the color change that occurred in the samples was statistically significant or not.

Results

Overall results showed that none of the archwires resisted color change after being immersed in staining solutions after two, four, and eight weeks, respectively, which was found to be statistically significant.

Conclusion

At the end of all time intervals, none of the archwires resisted a color change irrespective of the brand or coating of archwires. This result was found to be statistically significant. With respect to the solutions, all solutions from Chyavanprash, tea, coffee, vitamin C, turmeric milk, and AYUSH kadha displayed a staining effect on all the aesthetic archwires.

## Introduction

One of the prime reasons for patients seeking orthodontic treatment is improvement in their aesthetics or appearance. With a greater number of adult patients now opting for orthodontic treatment, the demand for aesthetic orthodontic materials has increased [1]. The patients desire to undergo orthodontic treatment without compromising their appearance during the treatment period. The increasing demand for more aesthetic orthodontic appliances has elicited an aesthetic revolution marked by the emergence of invisible appliances such as aesthetic brackets, lingual appliances, and clear aligners [2]. Ceramic brackets, clear aligners, and tooth-colored archwires are the new yardsticks for aesthetic orthodontic appliances. They are promising alternatives to conventional metallic materials that contain nickel for patients with nickel sensitivity [3]. Orthodontic archwires have significantly evolved from their original conception. Previously made of gold, they are currently made of various alloys such as stainless steel, nickel-titanium, copper-titanium, and other such alloys [4]. With the advent of ceramic and composite brackets, it was obvious for the archwires to change from their conventional metallic look to a more contemporary aesthetic look to enhance the patient’s appearance during the treatment period. The first esthetic transparent nonmetallic orthodontic wire known as Optiflex was made of a silica core, a silicone resin middle layer, and a stain-resistant nylon outer layer and was marketed by Ormco [5]. An archwire is usually replaced after four to eight weeks as per the schedule followed by orthodontists [6]. Thus, the wires need to sustain their aesthetic coating for at least 8 weeks before they are changed with the successive wire.

Based on preliminary research, only a few studies have been conducted (specifically in the American context). With the background of the recent coronavirus disease 2019 (COVID-19) pandemic and the popular role of strongly pigmented beverages that play an immunity-boosting role, studies exploring the effect of such beverages on orthodontic appliances may improve the decision-making process of selecting such aesthetic appliances [7].

## Materials and methods

**Figure 1:**
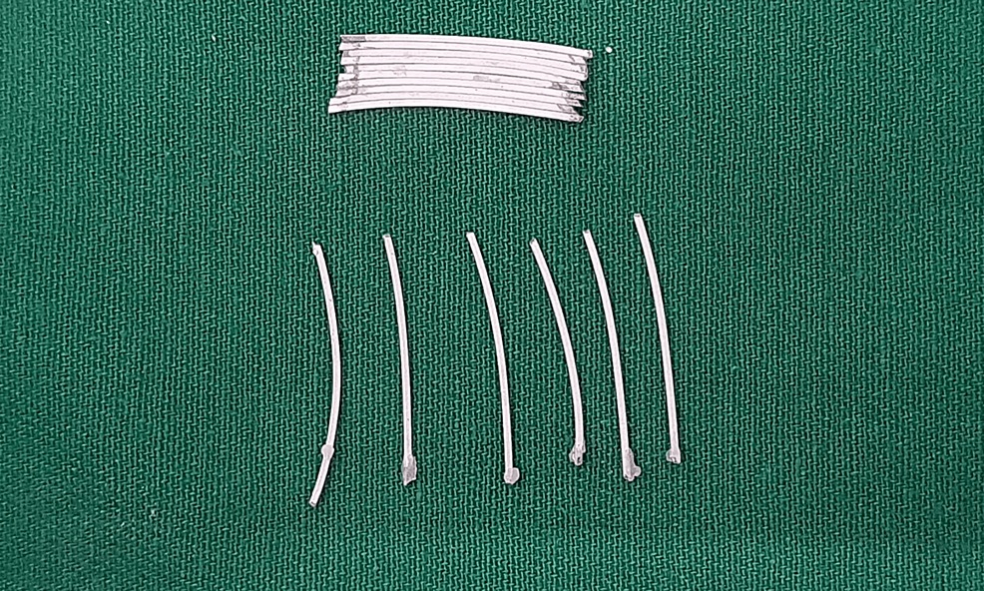
Preparation of samples by juxtaposing 10 mm long pieces of archwires

Four brands of wires were included in this study. The wires were Teflon-, epoxy-, or ceramic-coated. Convenience sampling was done, and five samples of each brand were prepared to be tested in each solution. The samples needed to be in a tile form of 10 x 10 mm dimension, as the minimum size required for spectrophotometry is 8 mm diameter. Archwires of one brand were marked and cut into equal pieces that were 10 mm long. The ends of these pieces were approximated such that light could not pass through them. The approximated pieces were kept on a glass slab coated with petroleum jelly to prevent them from sticking to the glass slab. The ends of the wires were glued together using light cure composite and glue as shown in Figure 1.

Three hundred ml of distilled water was used to prepare different solutions. After mixing and boiling, the solutions were cooled to room temperature and strained. A coffee solution was prepared using commercially available coffee powder (Nescafe) sachets. One teaspoon of coffee powder was added to 300 ml of boiling distilled water and stirred for uniform mixing as per the manufacturer’s instructions. Tea was prepared by adding commercially available tea powder. Two tablets of commercially available AYUSH kadha (Dhootapapeshwar dispersible tablet; Shree Dhootapapeshwar Limited, Punjab, India) were mixed as per instructions given by the manufacturer. Two vitamin C tablets (Limcee) were dissolved in 300 ml water and stirred well. A tablespoon of Chyavanprash was mixed in water at room temperature and stirred well. For making turmeric milk, one teaspoon of commercially available turmeric powder was added to boiling milk and allowed to cool.

All solutions were divided into four parts of 75 ml solution each using a measuring cylinder for staining four brands of archwire.

Before the specimens were immersed into the solution, the color of each sample was measured using the spectrophotometer and recorded as color at T0. The samples were immersed in their respective solutions for two weeks, four weeks, and eight weeks for 30 minutes each. Fresh solutions were supplemented every day.

Color measurement of the samples was done as follows.

Samples were tested at two, four, and eight weeks after immersing them in various solutions such as turmeric milk, AYUSH kadha, vitamin C tablets’ solution, coffee, Chyavanprash solution, and tea.

After the first measurement (T0), the samples were placed in a container with the prepared staining solution. Color measurements were repeated after two weeks(T1), four weeks (T2), and eight weeks (T3) of immersion in the solution. Before each measurement, samples were removed from the solution and rinsed with water. Excess water on the surfaces was blotted with tissue papers, and the samples were allowed to dry. Thereafter, the samples were subjected to spectrophotometric analysis. The spectrophotometer model used in this study was VITA Zahnfrabik H. Rauter GmbH & Co. KG, Germany, Sr No. H57127.

**Figure 2:**
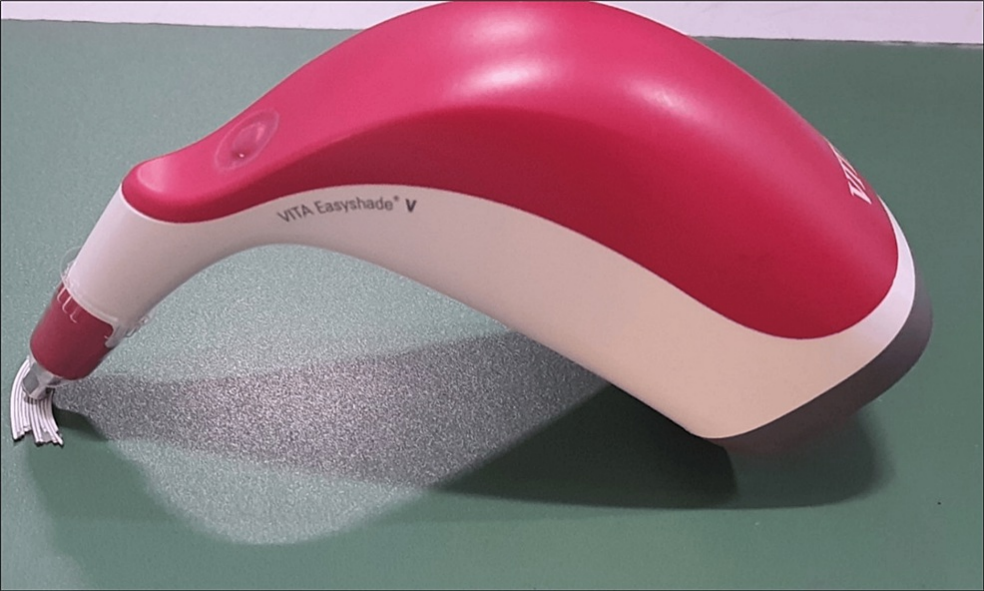
Measurement of color change using the spectrophotometer

The samples were placed on a flat surface with a green background. The nose of the spectrophotometer was placed perpendicular to the center of the sample (Figure 2). The spectrophotometer automatically generated three measurements from which it calculated a mean color measurement which was seen on the spectrophotometer’s screen. Color changes were characterized using the Commission Internationale de I’Eclairage L*a*b* color space (CIE L*a*b*).

The ΔE value of each sample was thus calculated.

Color differences (ΔE*) were determined using the following equation:

ΔE= [(ΔL)^2^ + (Δa)^2^ + (Δb)^2^]^ 1/2^

Where ΔE = color difference between the respective samples before and after the intervention.

ΔL = differences in the 'L.' value [darkness (0) or lightness (100)]

Δa = differences in the "a' value [redness (positive a*) or greenness (negative a*)]

Δb = differences in the b value [yellowness (positive b*) or blueness (negative b*)]

L*, a*, and b* values before (T0) and after immersion at each time interval (T1, T2, T3).

To relate the amount of color change (ΔE *) to a clinical environment, the data were converted to National Bureau of Standards (NBS) units as follows: NBS units =ΔE* x 0.92.

The definitions of color changes quantified by NBS units were used. These values were suggested by Koksal and Dikbas as shown in Table 1 [8].

**Table 1:** Critical marks of color change according to National Bureau Standards (NBS)

NBS Unit	Definitions of Color Differences	
0.0-0.5	Trace	Extremely slight change
0.5-1.5	Slight	Slight change
1.5-3	Noticeable	Perceivable change
3.0-6.0	Appreciable	Marked change
6.0-12.0	Much	Extremely marked change
12.0+	Very much	Change to other color

Statistics

A comparison of aesthetic degradation due to color changes among four brands of archwires was done by applying the one-way analysis of variance (ANOVA) test. The p values were calculated for all samples to determine whether the color change that occurred in the samples was statistically significant or not. Descriptive statistics were used to test the degree of color change using ΔE based on NBS units.

## Results

**Figure 3:**
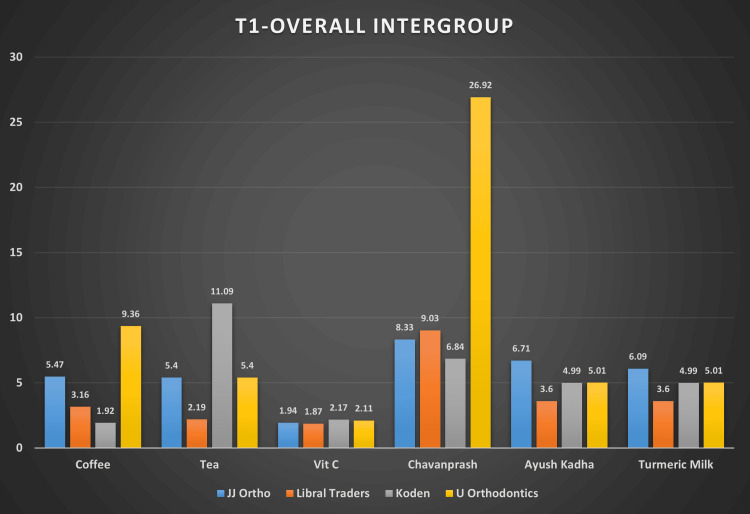
Bar graph showing mean color change (ΔE) values at T1 for four brands of wires in six solutions The data has been represented as the mean color change, i.e. (ΔE), at the first interval

Table 2 provides the descriptive statistics like mean and standard deviation for color change, i.e., ΔE obtained for four brands of wires dipped in six solutions for two weeks. At two weeks, the highest ΔE value of 26.92 (0.35) was noticed in the U Orthodontics (New Delhi, India) archwire after immersion in Chyavanprash solution (Table 2 and Figure 3). The least ΔE value of 1.87 (0.39) was observed in the Libral Traders (New Delhi, India) archwire group in a vitamin C solution (Table 2 and Figure 3).

**Table 2:** The overall intergroup comparison of color change (ΔE) of different brands of aesthetic archwires in various staining solutions at T1, i.e., two weeks The data is represented as change in color (ΔE) as Mean (SD = standard deviation), F = ratio of variance, p= p value such that p> 0.05 means no significant difference, *p< 0.05 means significant change and **p< 0.001 means a highly significant change in color.

T1	Coffee Mean (SD)	Tea Mean (SD)	Vit C Mean (SD)	Chyavanprash Mean (SD)	Ayush Kadha Mean (SD)	Turmeric Milk Mean (SD)
JJ Orthodontics	5.47 (1.12)	5.4 (0.42)	1.94 (0.57)	8.33 (0.76)	6.71 (0.5)	6.09 (0.44)
Libral Traders	3.16 (1.22)	2.19 (0.4)	1.87 (0.39)	9.03 (0.33)	3.6 (0.4)	3.6 (0.4)
Koden	1.92 (0.46)	11.09(0.49)	2.17 (0.4)	6.84 (0.23)	4.99 (0.15)	4.99 (0.15)
U Orthodontics	9.36 (0.25)	5.4 (0.16)	2.11 (0.28)	26.92 (0.35)	5.01 (0.15)	5.01 (0.15)
One-way ANOVA F test	F = 70.44	F = 441.19	F = 0.55	F = 2056.0	F =196.2	F =50.466
P value	p< 0.001**	p< 0.001**	p< 0.001**	p< 0.001**	p< 0.001**	p< 0.001**

Table 3 provides descriptive statistics like the mean and standard deviation for color change, i.e., ΔE, obtained for four brands of wire dipped in six solutions for four weeks. At four weeks, the color change intensified for all the archwires with a significant increase in ΔE.

**Table 3:** The table represents the overall intergroup comparison of the color change (ΔE) of different brands of aesthetic archwires in various staining solutions at T2 The data are represented as change in color (ΔE) as mean (SD = standard deviation), F = ratio of variance, p = p value such that p> 0.05 means no significant difference, *p< 0.05 means significant change, and **p< 0.001 means highly significant change in color.

T2	Coffee Mean (SD)	Tea Mean (SD)	Vit C Mean (SD)	Chyavanprash Mean (SD)	Ayush Kadha Mean (SD)	Turmeric Milk Mean (SD)
JJ Orthodontics	8.03 (0.63)	8.15 (0.36)	3.09 (0.27)	11.06 (0.26)	7.88 (0.34)	6.09 (0.26)
Libral Traders	4.96(0.31)	3.69 (0.4)	3.82 (0.32)	11.71 (0.23)	3.23 (0.07)	3.37 (0.18)
Koden	4.61 (0.32)	12.16 (0.23)	3.89 (0.27)	10.46 (0.31)	4.11 (0.15)	4.93 (0.19)
U Orthodontics	8.18 (0.43)	7.32 (0.25)	4.85 (0.21)	14.31 (0.26)	6.98 (0.23)	7.76 (0.12)
One-way Anova F test	F = 93.66	F = 577.68	F = 34.80	F = 195.96	F = 499.32	F = 437.24
P value	p< 0.001**	p< 0.001**	p< 0.001**	p< 0.001**	p< 0.001**	p< 0.001**

**Figure 4:**
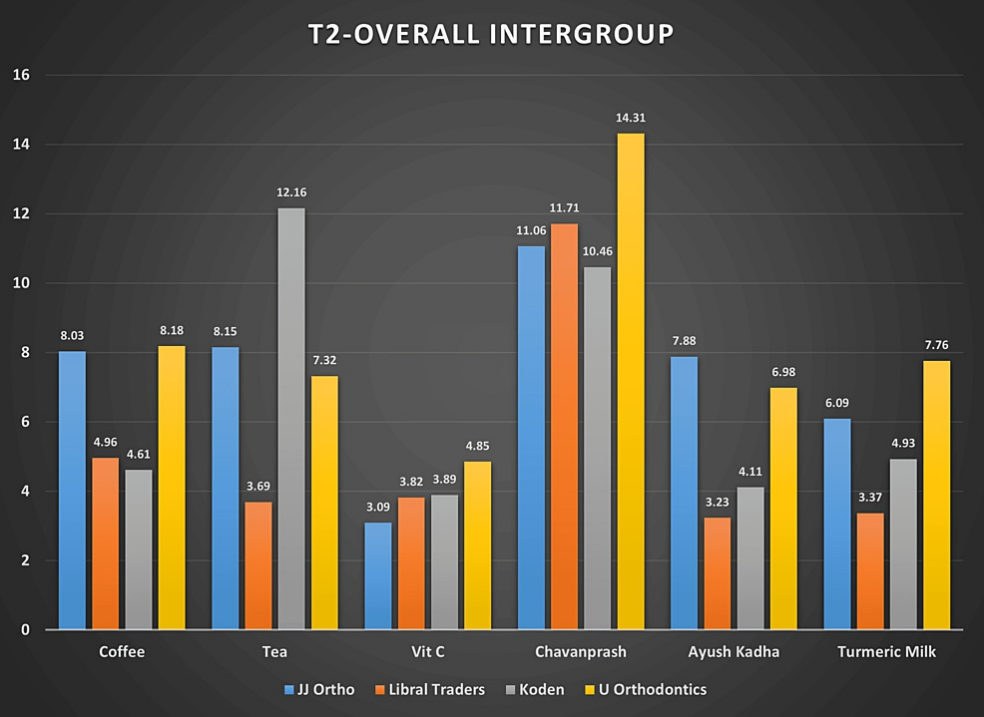
Bar graph showing the mean color change (ΔE) values at T2 for four brands of wires in six solutions The data have been represented as the mean color change, i.e., (ΔE), at the second interval.

JJ Orthodontics (Kerala, India) showed the ΔE 3.09 (0.27) in a vitamin C solution. Koden (Kerala, India) archwires showed more color change in a tea solution as compared to other archwires with ΔE 12.16 (0.23). Overall, the color change was less intense in the vitamin C solution and with Libral Traders archwires, whereas color change increased in the Chyavanprash solution and the U Orthodontics archwires (Figure 4).

The pairwise intergroup comparison at T2, i.e., four weeks, suggested that the difference in color change among various brands of archwires was statistically significant for most of the solutions. The result was statistically highly significant for all intergroup comparisons for AYUSH kadha, turmeric milk, Chyavanprash, and tea. There was no difference in color degradation between JJ Orthodontics and U Orthodontics archwires in the coffee solution. Libral and Koden had a similar amount of color change in the vitamin C solution as the p value was >0.05.

**Figure 5:**
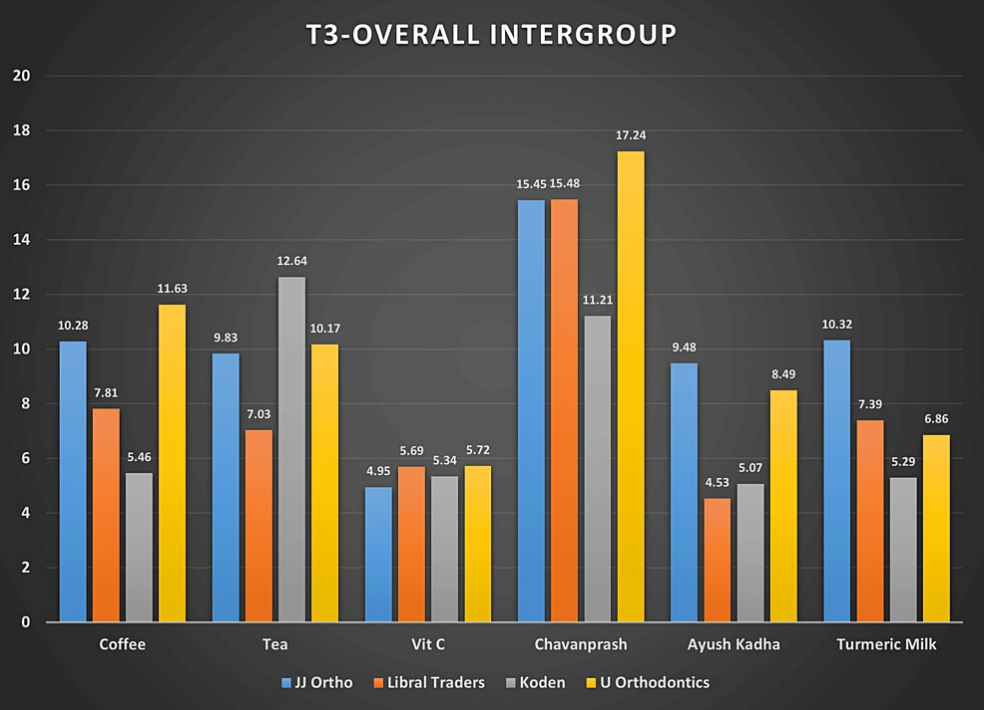
Bar graph showing mean color change (ΔE) values at T3 for four brands of wires in six solutions The data have been represented as the mean color change, i.e., (ΔE), at the third interval.

Table 4 provides the descriptive statistics like mean and standard deviation for color change, i.e., ΔE, obtained for four brands of wire dipped in six solutions for eight weeks (T3). At eight weeks, the color change intensified for all the archwires with a significant increase in ΔE (Figure 5). The color change was maximum in U Orthodontics archwires and Chyavanprash solution (Figure 5). The difference was statistically significant for all archwires in all solutions.

**Table 4:** Overall intergroup comparison of the color change of different brands of aesthetic archwires in various staining solutions at T3 The data are represented as the change in color (ΔE) as mean (SD = standard deviation), F = ratio of variance, p = p value such that p> 0.05 means no significant difference, *p< 0.05 means significant change, and **p< 0.001 means a highly significant change in color.

T3	Coffee Mean (SD)	Tea Mean (SD)	Vit C Mean (SD)	Chyavanprash Mean (SD)	Ayush Kadha Mean (SD)	Turmeric Milk Mean (SD)
JJ Orthodontics	10.28 (1.2)	9.83 (0.19)	4.95 (0.37)	15.45 (0.27)	9.48 (0.2)	10.32 (0.28)
Libral Traders	7.81 (1.81)	7.03 (0.38)	5.69 (0.29)	15.48 (0.23)	4.53 (0.19)	7.39 (0.43)
Koden	5.46 (0.86)	12.64 (0.32)	5.34 (0.23)	11.21 (0.4)	5.07 (0.2)	5.29 (0.22)
U Orthodontics	11.63 (4.03)	10.17 (0.09)	5.72 (0.37)	17.24 (0.33)	8.49 (0.33)	6.86 (0.2)
One-way ANOVA F test	F = 6.838	F=355.206	F = 6.044	F = 326.209	F=524.21	F= 242.29
P value	P=0.004*	p< 0.001**	P=0.006*	p< 0.001**	p< 0.001**	p< 0.001**

NBS values at the end of eight weeks suggested that almost all archwires showed ‘much’ difference in color. The vitamin C solution caused only appreciable color changes in the archwires as compared to the Chyavanprash solution, which led to ‘very much’ change.

All intergroup comparisons at the end of eight weeks (T3) indicated that changes produced by the vitamin C solution are not statistically significant for archwires. P value was <0.001 for all brand groups in the Chyavanprash solution except in JJ Orthodontics versus the Libral Traders group (Figure 5). Also, the color change among most groups of brands in the vitamin C solution was almost similar and thus statistically insignificant.

Overall results showed that none of the archwires resisted color change after being immersed in staining solutions after two, four, and eight weeks, respectively.

## Discussion

Multiple studies have been conducted on the color stability of various archwires in different staining solutions such as coffee, tea, cola, wine, etc. The consumption of beverages in the Indian context is quite different and has considerably changed after the COVID pandemic. As per the guidelines given by the AYUSH Department of the Government of India, it was recommended to drink AYUSH kadha and golden milk (turmeric milk) once or twice daily to boost immunity [7]. Chyavanprash, which is composed of a highly concentrated mixture of nutrient-rich plants and minerals, was also suggested by the same guidelines, as it intends to boost immunity [9]. As the ingredients of these beverages tend to have a staining effect, our study aimed whether the aesthetic archwires maintained their color on consistently encountering these staining solutions.

Usually, the duration between two appointments to change archwires is four to six weeks [6]. Previous studies by da Silvaa et al. (2013), Deepika S et al. (2016), and Anand A (2020) measured the color change after three weeks [10-12]. This is the minimum duration that wires should resist color change before they are replaced. Hence, the time intervals of two, four, and eight weeks (T1, T2, and T3, respectively) were selected for our study.

Of the four brands of wires used in this study, two had Teflon coating (JJ Orthodontics and U Orthodontics) while one had ceramic coating (Koden) and one had epoxy coating (Libral Traders). Results showed that irrespective of the brand and coating, all archwires displayed a staining effect when immersed in different solutions. The finding that epoxy-coated archwires were more color-stable than Teflon-coated wires is consistent with the findings of the study conducted by Anand A (2018) who used red wine, orange juice, and mouthwash as staining solutions [12]. JJ Orthodontics showed the minimum color change as per the ΔE values in vitamin C as compared to the other wires. U Orthodontics showed less staining (6.86) than Libral Trades (7.39) in turmeric milk. U Orthodontics wires, which were Teflon-coated, showed maximum staining among all the archwires followed by JJ Orthodontics, which were also Teflon-coated. Libral Traders’ archwires resisted staining the most followed by Koden archwires. Thus, epoxy coating and ceramic coating seemed to have better stain resistance as compared to other coatings. Studies conducted by Anand A et al., Alsanea et al., and Ismail N et al. had similar conclusions [12-14]. Teflon-coated wires were more stained even when immersed in fluoridated and non-fluoridated mouthwashes as per the study by Hussein L et al. [15]. As compared to rhodium coating, epoxy-coated archwires had an almost equal color change value. Whereas rhodium archwires were superior to Teflon-coated archwires in maintaining their color stability [14]. According to all these experiments, the Teflon-coated aesthetic archwires were more prone to color changes when dipped in various dietary staining solutions. Teflon-coated archwires' higher propensity for a color shift may be caused by the production process [16].

With respect to the solutions (i.e. Chyavanprash, tea, coffee, turmeric milk, vitamin C solution, and AYUSH Kadha) included in this study, ΔE was observed over two, four, and eight weeks. Some studies conducted on ceramic brackets concluded that wine was the most staining solution in comparison with other staining substances such as mouthwash and cola drinks [12]. Studies by Mutlu-Sagesen L et al. and Ertaş E et al. concluded that coffee was the most staining solution in comparison with other staining substances such as tea and cola drinks [17-18]. Ismail N et al. suggested that adding milk to the preparation reduced the staining effect of coffee and tea and may reduce the concentration of staining pigments present in these solutions [14]. The studies mentioned above support the use of such solutions to test the color stability of archwires.

Although the present study did not statistically test the highest staining agent, vitamin C tablets dissolved in distilled water were observed to have a lower staining effect followed by AYUSH kadha tablet solution according to the ΔE findings. The reason for this observation could be that both these tablets were completely dispersible and thus showed minimum remnants on the wire surface and could be easily cleaned with tap water.

Since none of the previous studies included Chyavanprash solution, AYUSH kadha, turmeric milk, or vitamin C solution, their effect on archwires was a novel finding in the study. The staining of archwires was visible to the naked eye at all time intervals for all solutions, suggesting that none of the archwires could be the yardsticks for good aesthetic materials.

Limitations

This study explored the color stability of four brands of aesthetic archwires in six beverages. However, it also has some limitations that provide scope for future research. For example, more brands of wires can be incorporated in such a study. The in vivo evaluation of the color stability of archwires should be evaluated as the environment of the oral cavity may have a different effect on the staining of archwires. However, it may not be possible to monitor the effect of a single solution on an archwire if the study is conducted intraorally as other factors such as the patient’s oral hygiene and salivary flow can change the results.

Clinical significance

Based on the current research, it can be concluded that currently, epoxy-coated archwires could be preferred for a patient undergoing fixed orthodontic therapy with aesthetic brackets. With the background of the COVID-19 pandemic, vitamin C and Ayush kadha are suitable for the aesthetic preservation of archwires.

## Conclusions

Our study tested the staining effect of six solutions on four different brands of archwires at two, four, and eight weeks. At the end of all time intervals, none of the archwires resisted a color change irrespective of the brand or coating of archwires. With respect to the solutions, all solutions, i.e. Chyavanprash, tea, coffee, vitamin C, turmeric milk, and AYUSH kadha, displayed a staining effect on all the aesthetic archwires. ΔE values suggest that there could be a difference in the degree of color change in the various staining solutions, the statistical significance of which can be investigated in future studies.

Since the consumption of beverages apart from tea and coffee used in this study is becoming popular worldwide, the results of this study can be implicated not only in the Indian context but also globally. Overall, this study provides a basis for further research, which includes more solutions and archwires to statistically determine the most aesthetically stable archwires. This can help clinicians guide their patients better in maintaining the aesthetics of their appliances throughout the treatment.
